# Human adenovirus type 19 infection of corneal cells induces p38 MAPK-dependent interleukin-8 expression

**DOI:** 10.1186/1743-422X-5-17

**Published:** 2008-01-25

**Authors:** Jaya Rajaiya, Jingnan Xiao, Raju VS Rajala, James Chodosh

**Affiliations:** 1 Molecular Pathogenesis of Eye Infection Research Center, Dean A. McGee Eye Institute, Department of Ophthalmology, University of Oklahoma Health Sciences Center, Oklahoma City, Oklahoma, USA

## Abstract

**Background:**

Human adenovirus type 19 (HAdV-19) is a major cause of epidemic keratoconjunctivitis, the only ocular adenoviral infection associated with prolonged corneal inflammation. In this study, we investigated the role of p38 mitogen-activated protein kinase (MAPK) in HAdV-19 infection, with particular attention to the role of p38 MAPK in the transcriptional control of interleukin-8 (IL-8), a chemokine previously shown to be central to the initiation of adenovirus keratitis.

**Results:**

We found that infection of corneal cells with HAdV-19 led to activation of p38 MAPK and its downstream targets, HSP-27 and ATF-2, within 15 to 30 minutes post-infection. Infection also induced phosphorylation of IκB and NFκB in a p38 MAPK-dependent fashion. Furthermore, HAdV-19 induced an interaction between p38 MAPK and NFκB-p65, followed by nuclear translocation of activated NFκB-p65 and its binding to the IL-8 promoter. The interaction between p38 MAPK and NFκB-p65 was inhibited in concentration-dependent fashion by SB203580, a chemical inhibitor of p38 MAPK, but not by SP600125, an inhibitor of JNK – another MAPK implicated in chemokine expression by HAdV-19 infected cells. IL-8 gene expression in HAdV-19 infection was significantly reduced in the presence of sequence-specific p38 MAPK siRNA but not control siRNA.

**Conclusion:**

These results provide the first direct evidence for transcriptional regulation of IL-8 in HAdV-19 infected cells through the activation of the p38 MAPK signaling pathway. The p38 MAPK pathway may play a biologically important role in regulation of IL-8 gene expression in the adenovirus-infected cornea.

## Background

Epidemic keratoconjunctivitis is an explosive and highly contagious ocular surface infection associated with prolonged corneal stromal inflammation [1], and caused by species D human adenovirus (HAdV) serotypes 8, 19, and 37 [2]. After contact between the adenoviral capsid fiber knob with one of several potential primary adenovirus receptors [3], a secondary interaction between more proximal arginine-glycine-aspartic acid amino acid sequences in the adenoviral penton capsomer and target cell integrins α_v_β_3 _and α_v_β_5 _[4,5] induces activation of an intracellular signaling cascade involving Src family kinases, phosphoinositide 3-kinase (PI3K), and Rho family GTPases, which in turn leads to actin polymerization and clathrin mediated endocytosis of the virus [6,7]. While internalization of the virus is unequivocally mediated by an intracellular signaling cascade, other consequences of intracellular signaling upon HAdV-19 infection of corneal cells were more recently reported, including PI3K/Akt-mediated promotion of cell viability during viral replication [8], and Src kinase-dependent expression of pro-inflammatory mediators [9].

The mitogen-activated protein kinases (MAPKs) integrate a wide range of upstream signals to determine patterns of downstream gene expression through the regulation of transcription factors. The ERK1/2, p38, and JNK MAPK pathways have been well characterized. The p38 MAPK signaling cascade regulates numerous cellular functions, including the cell cycle, development, differentiation, apoptosis, and inflammation, dependent on the specific cell type and extracellular stimulus [10]. In inflammation, activation of the p38 MAPK superfamily is critical to the conversion of external stimuli to pro-inflammatory gene expression [11], and reportedly impacts the expression of IL-8, IL-6, ICAM-1 [12-14], COX-2, and PGE2 [15]. Four isoforms of p38 MAPK, α, β, γ, and δ, are expressed in a cell specific manner [10], with the ubiquitously expressed α isoform most prominently implicated in cytokine production [16,17].

IL-8 is a C-X-C chemokine that induces chemotaxis of various cell types, particularly neutrophils [18], and is induced by a variety of stimuli, including tumor necrosis factor, IL-1, bacterial and viral infection [19]. Transcriptional regulation of IL-8 has been extensively studied, and the NFκB transcription factor family is believed to play a central role [20]. NFκB in the cytoplasm exists as subunit homodimers (e.g., p50p50 and p65p65) and heterodimers (p50p65) bound to the inhibitor of κB (IκB). With the appropriate stimulus, IκB kinase initiates phosphorylation and degradation of IκB, thus freeing NFκB to form transcriptionally active complexes that translocate to the nucleus [21-23]. In the nucleus, specific NFκB dimers bind specific promoters for transcriptional activation [20]. Inhibitors of ERK and p38 MAPK each attenuated the activation of NFκB in glomerular cells [24]. However, an interaction between p38 MAPK and NFκB has not been explored, and the potential role of p38 MAPK in the transcriptional regulation of IL-8 in corneal cells remains unknown.

The eye represents a major target of adenovirus infection, and the resultant inflammation, particularly in the HAdV-19-infected cornea, can lead to long term aberrations in vision and comfort [1]. The means by which HAdV-19 infection of the eye induces corneal cells to express specific chemokines is not fully understood. As the predominant cell type within the corneal stroma, fibroblast-like keratocytes play a major role in defense against pathogens and injury caused to the cornea, having been shown to express numerous pro-inflammatory mediators, including IL-1, IL-6, IL-8, MCP-1, TNF-α, RANTES, and G-CSF [25-27]. HAdV-19 infection of keratocytes induces IL-8 expression, and it has been suggested that subsequent binding and persistent maintenance of the IL-8 signal within corneal extracellular matrix plays a major role in the chronic and recurrent corneal stromal inflammation associated with infection [9,28]. We have previously shown that HAdV-19 infection of keratocytes results in activation of focal adhesion kinase, cSrc, ERK, and JNK, and that these signaling proteins assist in the expression of inflammatory mediators from virus-infected cells [9,29,30]. Because the activation of p38 MAPK and its downstream signaling pathway appears to be central to IL-8 expression, we studied the role of p38 MAPK in HAdV-19 infection of keratocytes and their subsequent expression of IL-8. Herein, we show that infection of keratocytes with HAdV-19 activates the p38 MAPK signaling pathway, and induces p38 MAPK-specific activation and nuclear translocation of NFκB, possibly through an interaction between p38 MAPK and NFκB-p65. NFκB activation and IL-8 expression at both mRNA and protein levels proved to be p38 MAPK-dependent.

## Results

### HAdV-19 infection activates p38 MAPK

**Figure 1:**
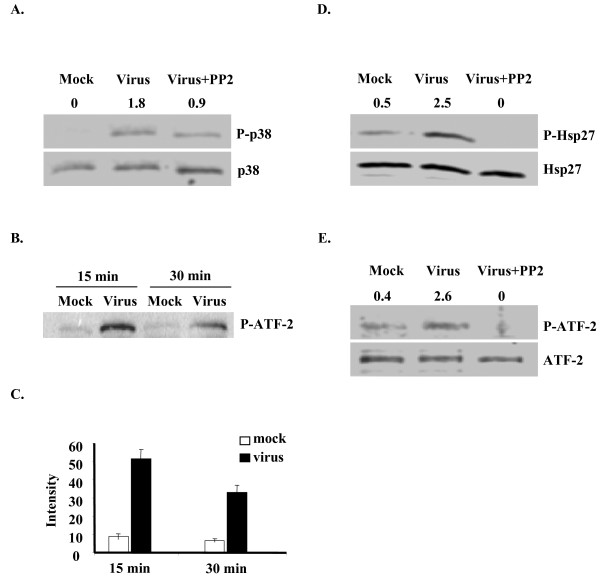
**HAdV-19 infection of keratocytes induces activation of p38 MAPK**. Lysates were prepared 30 min after HAdV-19 or mock infection. (A) Western blot analysis using antibodies against phospho- and total p38 MAPK reveals phosphorylation in HAdV-19 infected cells, reduced in the presence of the Src inhibitor PP2 (10 μM). Densitometry values for the phosphorylated protein (normalized to the corresponding total protein) are shown above each lane. (B) An *in vitro *p38 MAPK assay performed at 15 and 30 min after infection shows increased phosphorylation of the ATF-2 substrate signifying p38 MAPK activity upon HAdV-19 infection of keratocytes. (C) Densitometric quantification from three experiments of the phosphorylated ATF-2 band in the p38 MAPK assay revealed increased activity in virus infected keratocytes at both 15 and 30 min post-infection (p = 0.0012 and p = 0.0025, respectively). (D & E) Western blot analysis using phospho- and total antibodies against HSP27 and ATF-2 respectively, reveals increases in phosphorylation in HAdV-19 infected cells that was absent in the presence of PP2.

To determine whether adenovirus infection induced the activation of p38 MAPK, we infected keratocytes with HAdV-19 and immunoblotted for phosphorylation of p38. The results indicate increased phosphorylation of p38 MAPK compared to mock infection (Fig. 1A). To further determine whether HAdV-19 infection regulates the kinase activity of p38 MAPK, we performed a kinase assay using exogenous GST-ATF-2 fusion protein as a substrate. HAdV-19 infection induced an increase in the ability of p38 MAPK to phosphorylate exogenous ATF-2 substrate at both 15 and 30 min post-infection as compared with p38 MAPK from mock infected cells (Fig. 1B). Quantification revealed approximately 5-fold more p38 MAPK activity in virus infected keratocytes at 15 and 30 minutes post-infection, respectively (Fig. 1C; p = 0.0012 at 15 minutes and p = 0.0025 at 30 minutes). These data suggest that p38 MAPK is activated in HAdV-19 infection. To further determine whether HAdV-19 infection specifically induces the activation of p38 MAPK alone or the complete MAPK pathway, we examined two downstream effectors of p38 MAPK, HSP27 and ATF-2. The results indicate that HAdV-19 infection increased the phosphorylation of HSP27 (5-fold) and ATF-2 (6.5-fold) over mock infection (Fig. 1D and 1E). These results suggest that HAdV-19 infection activates the p38 MAPK pathway.

We have previously reported that HAdV-19 infection resulted in the activation of Src and further demonstrated that Src kinase inhibitor blocked the induction of inflammatory chemokine IL-8 in keratocytes [9]. To determine whether Src is upstream or downstream of p38 MAPK, we examined the HAdV-19 induced activation of p38 MAPK in the presence and absence of Src kinase inhibitor, PP2. As shown, PP2 decreased the activation of p38 MAPK (Fig. 1A). PP2 also completely blocked the activation of HSP27 and ATF-2 (Fig. 1D and 1E). Furthermore, PP2 reduced overall p38 MAPK activity in HAdV-19 infected cells (data not shown). Collectively these data suggest in HAdV-19 infection, that p38 MAPK is downstream of Src kinase.

### p38 MAPK specific NFκB-p65 activation in HAdV-19 infected keratocytes

**Figure 2:**
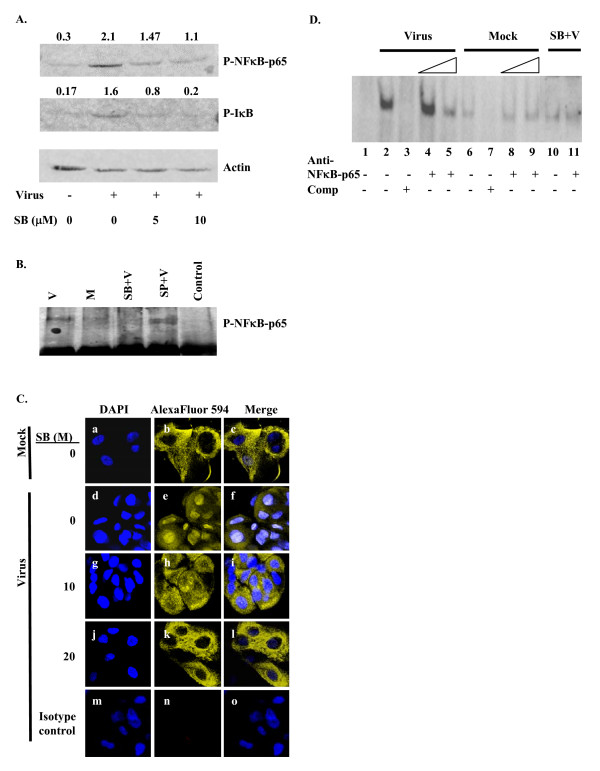
**p38 MAPK-dependent NFκB-p65 activation in HAdV-19 infected keratocytes**. Cells were incubated with the p38 MAPK inhibitor SB203580 (5 or 10 μM) prior to HAdV-19 or mock infection, and lysates prepared 30 min after infection. (A) NFκB-p65 and IκB phosphorylation increased with virus compared to mock infection, and were reduced in cells pre-treated with SB203580 in dose-dependent fashion. Densitometry values for the phosphorylated protein (normalized to the corresponding actin levels) are shown above each lane. (B) Immunoprecipitation assay reveals association of p38 MAPK with NFκB-p65 in virus infected (V) but not in mock (M) infected cells. This association was blocked by the p38 MAPK inhibitor SB203580 (SB) but not by the JNK inhibitor SP600125 (SP). Isotype control did not immunoprecipitate any NFκB-p65. (C) NFκB-p65 activation in HAdV-19 infection analyzed by confocal microscopy. The left column shows DAPI staining for nuclei (blue), the middle column for p65 (yellow), and the right column a merging of the left 2 rows. (c) Mock infected keratocytes show mostly cytoplasmic localization of NFκB-p65. (f) HAdV-19 infected keratocytes at 20 min post-infection show nuclear localization of NFκB-p65. (i) Nuclear translocation of NFκB-p65 was reduced in the presence of 10 μM, and (l) completely blocked with 20 μM SB203580. Bottom row (m, n, o), represent isotype control. (D) Electromobility shift assay showing NFκB-p65 binding to IL-8 promoter in HAdV-19 infected keratocytes. Extracts from HAdV-19 infected cell nuclei show more binding to NFκB-specific IL-8 probe (lane 2) as compared to nuclear protein from mock infected cells (lane 6) or when pretreated with SB203580 (lane 10). Binding specificity of the probe is shown with 100 molar excess of unlabelled probe (lanes 3 and 7). SB203580 (SB) blocked NFκB binding in virus infected nuclear extracts (lane 10). Dose dependent supershifts using increasing amounts of NFκB-p65 antibody are shown in virus infected nuclear extracts (lanes 4 and 5). No shifts were observed in nuclear extracts from mock infected cells (lanes 8 and 9) or SB203580 treated cells (lane 11).

NFκB is a molecule well known to play a crucial role in IL-8 gene expression [31-35]. We have reported previously increased amounts of NFκB-p65 in the nuclear extracts from HAdV-19 infected keratocytes [8]. The relationship between p38 MAPK activation and the nuclear localization of NFκB-p65 in HAdV-19 infection is not known. To determine the effect of p38 MAPK activation on the phosphorylation of NFκB-p65, we carried out experiments in the presence and absence of the p38 MAPK inhibitor, SB203580. The results indicate that HAdV-19 infection enhanced the phosphorylation of both IκB and NFκB-p65, and that increasing concentrations of p38 MAPK inhibitor resulted in a concentration-dependent inhibition of their phosphorylation (Fig. 2A). These results suggest that p38 MAPK regulates the activation of NFκB-p65.

### Interaction between p38 MAPK and NFκB-p65 in HAdV-19 infection

To determine whether p38 MAPK interacts with NFκB-p65, p38 MAPK immunoprecipitates from mock and virus infected cells were subjected to SDS PAGE and Western blot analysis with anti-phospho-NFκB-p65. The results indicate an interaction between NFκB-p65 and p38 MAPK protein in HAdV-19 infected keratocytes (Fig. 2B). We observed no such interaction in mock infection. The interaction between NFκB-p65 and p38 MAPK protein was abolished in the presence of the p38 MAPK inhibitor SB203580. In contrast, the JNK pathway inhibitor SP600125 failed to affect the interaction (Fig. 2B). These results clearly suggest the specificity of p38 MAPK activation in its functional association with NFκB-p65.

### p38 MAPK dependent nuclear translocation of NFκB-p65 in HAdV-19 infection

Our *in vitro *experiments clearly suggest a HAdV-19-induced interaction between NFκB-p65 and p38 MAPK (Fig. 2B). However, nuclear translocation of NFκB is necessary for its function. To determine whether p38 MAPK influences NFκB-p65 nuclear translocation, we examined the nuclear localization of NFκB-p65 in HAdV-19 infection in the presence or absence of p38 MAPK inhibitor by confocal microscopy. Nuclear localization of NFκB-p65 was visualized at 20 min post infection in HAdV-19 infected cells (Fig. 2C (f)), while mock infected cells showed mostly cytoplasmic staining (Fig. 2C (c)). The p38 MAPK inhibitor SB203580 inhibited NFκB-p65 nuclear translocation in a concentration-dependent manner (Fig. 2C (i, l)). These results clearly suggest that p38 MAPK activation regulates association (Fig. 2B) and nuclear translocation (Fig. 2C) of NFκB-p65.

### Infection-dependent binding of NFκB to the IL-8 promoter

By electrophoretic mobility shift assay (EMSA), we observed an increased binding of NFκB to IL-8 promoter sequence in HAdV-19 infected keratocyte nuclear extracts (Fig. 2D, lane 2 versus lane 6). This binding was supershifted by anti-NFκB-p65 antibody in a dose-dependent fashion (lanes 4 and 5). Reduced binding and no supershift was observed in mock infected cells (lanes 8 and 9). Both binding of NFκB-p65 and its supershift were reduced in cells that were SB203580 treated before HAdV-19 infection (lanes 10 and 11, respectively). Specificity of probe binding was shown by use of 100 molar excess of unlabelled probe (lanes 3 and 7). NFκB-p65 protein levels were significantly higher in the nuclear extracts of virus infected keratocytes, and the increase was abrogated with mock infection or SB203580 pretreatment (data not shown). These data suggest that IL-8 induction due to HAdV-19 infection of keratocytes is mediated by the p38 MAPK-dependent binding of NFκB to the IL-8 promoter.

### p38 MAPK-dependent IL-8 expression in HAdV-19 infection

**Figure 3:**
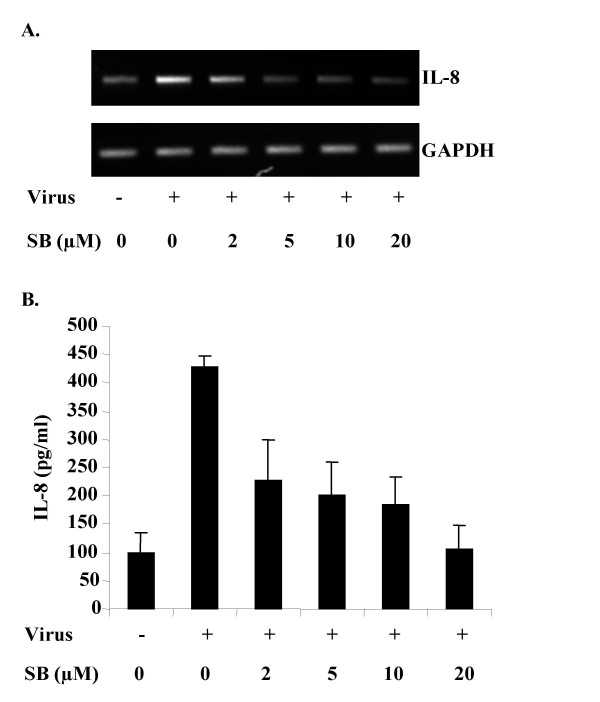
**HAdV-19-induced IL-8 expression**. (A) Increased IL-8 mRNA was detected by RT-PCR in HAdV-19 infected keratocytes, and this increase was reduced by pre-treatment with SB203580 (SB: 2, 5, 10, or 20 μM). GAPDH mRNA levels are shown as a control. (B) At 4 hr after infection, IL-8 protein was also significantly increased by ELISA, and this increase was reduced in dose-dependent fashion by SB203580 (p = 0.0041). Error bars represent the standard error of the mean. The figure shown represents four independent experiments.

Results from our study clearly suggest that HAdV-19 infection regulates p38 MAPK-dependent activation of NFκB and its binding to the IL-8 promoter (Fig. 2). To determine a functional relationship between IL-8 expression and p38 MAPK activation, we examined IL-8 transcription using RT-PCR and IL-8 protein expression by ELISA. Semi-quantitative RT-PCR results suggested that IL-8 transcription was enhanced in HAdV-19 infection, but reduced in the presence of the p38 MAPK inhibitor SB203580 (Fig. 3A). Quantitative real-time RT-PCR performed to measure IL-8 mRNA levels in HAdV-19 infected cells showed a 5-fold increase in IL-8 mRNA compared to mock-infected cells but only a 0.7-fold change in cells treated with 20 μM SB203580 prior to infection with HAdV-19 (data not shown). By ELISA, HAdV-19 infection for 4 hours increased IL-8 protein by approximately 5-fold compared to mock infected cells, while 20 μM SB203580 reduced IL-8 expression to mock infected levels (Fig. 3B). Regression analysis showed that the amount of IL-8 produced was inversely proportional to the concentration of SB203580 (p = 0.0041). These results suggest that IL-8 gene expression is p38 MAPK-dependent.

### p38 MAPK siRNA down regulation of IL-8 induction in HAdV-19 infection

**Figure 4:**
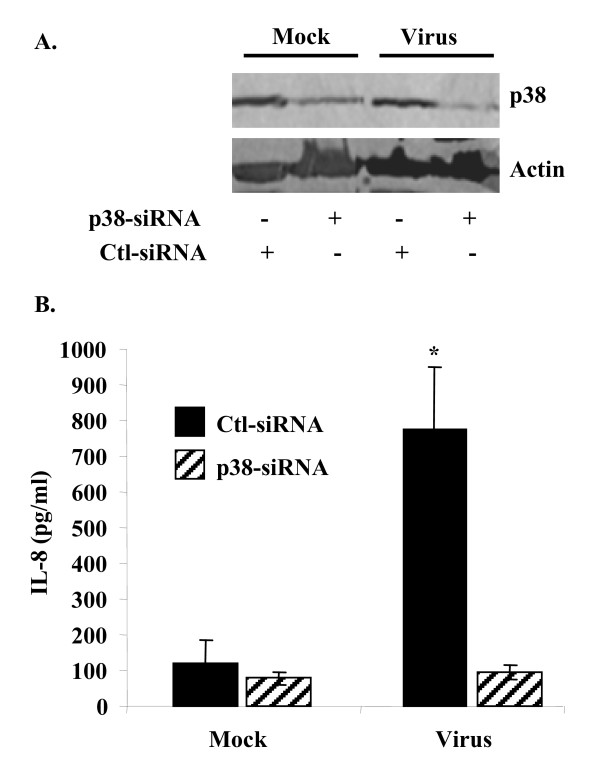
**p38 MAPK is required for IL-8 expression in HAdV-19 infected keratocytes**. (A) Cells transfected with p38 MAPK-specific siRNA show approximately 90% reduction in total p38 MAPK protein expression as compared to control siRNA in both mock and virus infected cells. (B) IL-8 production in virus infected keratocytes was reduced to mock infected levels by p38 MAPK-specific siRNA but not by control siRNA (*p = 0.0009).

To further confirm the biological significance of p38 MAPK activation in HAdV-19 infection, we knocked down p38 MAPK protein using p38 MAPK sequence-specific siRNA. and then HAdV-19 or mock infected the cells for 4 hours prior to ELISA for IL-8 expression. Densitometric analysis of Western blots for p38 MAPK protein expression revealed an approximately 90% reduction in total p38 MAPK after transfection with sequence-specific siRNA as compared with control siRNA. (Figure 4A). IL-8 ELISA performed on siRNA treated cells at 4 hours after infection showed a significant reduction in IL-8 expression in the p38 MAPK siRNA-treated cells (p = 0.0009) (Figure 4B). These results suggest that p38 MAPK activation may promote inflammation in HAdV-19 infected tissues through the regulation of IL-8 expression.

## Discussion

Ocular adenovirus infection begins with attachment of its penton fiber knob to a primary receptor such as the coxsackie-adenovirus receptor, sialic acid, or CD46 [36-38]. Secondary interaction between RGD sequences in the penton base protein and cellular integrins α_v_β_3_, α_v_β_5_, and others lead to integrin clustering [37], and subsequent virus internalization via phosphoinositide-3 kinase (PI3K) [39] and Rho family GTPase-dependent signaling [6]. In the human cornea, HAdV-19 infection leads to infiltration of leukocytes, presumably in response to chemokines produced by infected corneal cells [28]. *In vitro*, HAdV-19 infection of corneal cells leads to the activation of intracellular signaling cascades that induce chemokine expression [9,29,30]. MAPKs play a critical role in the assimilation and processing of external signals into key cellular functions, including inflammation [40,41], and are therefore likely integrators of upstream signaling in adenovirus infection. We previously reported that HAdV-19 binding to keratocytes activates the MAPK ERK1/2, and that its activity is necessary for subsequent IL-8 expression [9]. It has been previously shown that both ERK and p38 MAPKs are necessary for expression of the C-X-C chemokine IP-10 in HAdV-5 infected epithelial cells [42]. Furthermore, nuclear translocation of NFκB was required for IP-10 expression [43]. HAdV-3 induction of the C-C chemokine RANTES also requires nuclear translocation of NFκB [44]. In this context, we show for the first time that adenovirus infection induces an interaction between p38 MAPK and the NFκB-p65, and that this interaction requires p38 activation. We speculate that the p38 MAPK/NFκB-p65 interaction may promote the translocation of activated NFκB-p65 to the nucleus to bind to the IL-8 promoter, thereby directly inducing IL-8 gene expression. Our findings also suggest that the p38 MAPK pathway is activated downstream of Src kinase in HAdV-19 infection, as are ERK and JNK [9,30]. The p38 MAPK signaling cascade has previously been shown to promote microtubule-mediated nuclear targeting of HAdV-2 [45], and may regulate alternate splicing of adenoviral transcripts [46], suggesting a myriad of downstream actions of p38 MAPK signaling in adenovirus infection.

Our results suggest that IL-8 transcription in HAdV-19 infection in keratocytes is directly dependent upon the activation of p38 MAPK and NFκB. Both p38 MAPK inhibitor and sequence specific p38 MAPK siRNA reduced IL-8 protein expression in HAdV-19 infection, similar to *Listeria monocytogenes *infection, in which IL-8 production is mediated through a p38 MAPK-NFκB-p65 pathway [35]. In contrast, MCP-1 mRNA and protein expression, previously demonstrated to be under the regulation of the JNK cascade in HAdV-19 infection [30], were not reduced by the p38 MAPK inhibitor SB203580 (Rajaiya and Chodosh, unpublished data),. Taken together, these findings suggest that p38 MAPK activation is important for IL-8 but not MCP-1 expression in adenovirus-infected keratocytes. Src-dependent activation of ATF-2 and HSP27, downstream targets of p38 MAPK, was also observed. Activation of the transcription factor ATF-2 in the p38 MAPK pathway for IL-8 production was previously reported [47,48]. HSP27 phosphorylation was earlier shown to stimulate polymerization of actin [49,50]. Overexpression of HSP27 in melanoma cells reduced their migration [51] but increased migration of several other types of cells [52,53], suggesting that HSP27 has cell-specific functions. In HAdV-2 infection of HeLa cells, HSP27 appeared to assist in nuclear targeting of the virus [45], but its specific function in HAdV-19 infected corneal cells remains uncertain.

We demonstrated a physical association between p38 MAPK and the p65 subunit of NFκB in HAdV-19 infected cells, which was dependent upon p38 MAPK activation. Interestingly, nuclear translocation of NFκB-p65 as result of HAdV-19 infection was attenuated by p38 MAPK inhibitor. We do not yet know whether the association between p38 MAPK and NFκB-p65 was direct or mediated by a third protein. We also recently observed that downregulation of NFκB-p65 expression in keratocytes by NFκB-p65 specific siRNA results in significantly reduced activity of IL-8 promotor-driven luciferase, when driven by an IL-8 promoter containing NFκB binding sites (Rajaiya and Chodosh, unpublished data). Collectively, our studies suggest that in adenovirus infected keratocytes, p38 MAPK is activated for IL-8 induction through the IκB/NFκB pathway. This pathway is likely to be one of several responsible for the inflammatory phenotype in adenovirus-induced epidemic keratoconjunctivitis. Translational studies will be required to confirm the importance of p38 MAPK in the robust inflammatory response to HAdV-19 infection in human patients.

**Figure 5:**
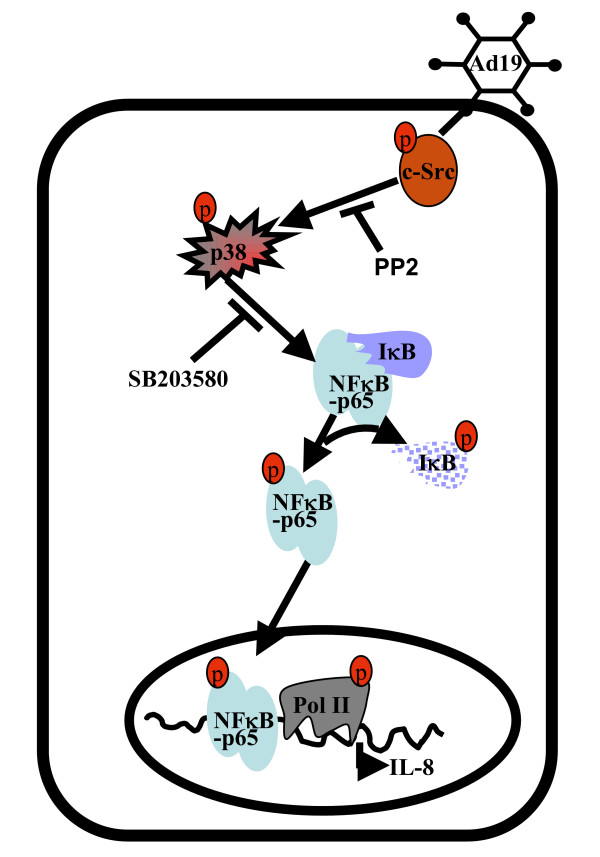
**Model for the role of p38 MAPK in adenovirus infected keratocytes**. Upon infection, p38 MAPK is phosphorylated in a Src-dependent fashion. p38 MAPK then phosphorylates IκB resulting in NFκB-p65 activation. Activated NFκB-p65 translocates to the nucleus where it binds specifically to the IL-8 promoter to transactivate IL-8 gene expression.

Based on our results, a working model is proposed (Fig. 5) in which HAdV-19 infection of keratocytes initiates a signaling cascade that involves activation of the nonreceptor tyrosine kinase c-Src, followed by p38 MAPK activation and NFκB-p65 nuclear translocation, leading ultimately to IL-8 gene expression. We speculate that compounds that modulate intracellular signaling in adenovirus infection might reduce the inflammatory component of infection. Understanding the signaling repertoire in HAdV-19 infection of keratocytes may elucidate the mechanisms behind corneal stromal inflammation in the disorder and represents a long term goal of our laboratory.

## Conclusion

These data show that infection of corneal cells with HAdV-19 leads to activation of p38 MAPK and its downstream targets within 15 to 30 minutes post-infection, and induces an interaction between p38 MAPK and NFκB-p65, followed by nuclear translocation of activated NFκB-p65 to bind to the IL-8 promoter. IL-8 gene expression in HAdV-19 infection was significantly reduced in the presence of sequence-specific p38 MAPK siRNA. Transcriptional regulation of IL-8 in HAdV-19 infected cells appears to occur through the activation of the p38 MAPK signaling pathway, suggesting a biologically important role in regulation of IL-8 gene expression in the adenovirus-infected cornea.

## Methods

### Reagents

Antibodies to p38 MAPK, ATF-2, HSP27, NFκB-p65, IκB, phospho-p38 MAPK, phospho-ATF-2, phospho-HSP27, phospho-NFκB-p65 and phospho-IκB were obtained from Cell Signaling Technology (Beverly, MA) and Santa Cruz Biotechnology (Delaware, CA). The anti-human IL-8 antibody and biotin-conjugated anti-human IL-8 antibody were from BD PharMingen (San Diego, CA). The c-Src inhibitor PP2 and p38 MAPK inhibitor SB203580 were purchased from Calbiochem (La Jolla, CA).

### Cell culture and viruses

Primary keratocytes were derived from donor corneas as previously described [30]. Briefly, after mechanical debridement of the corneal epithelium and endothelium, corneas were cut into 2 mm-diameter sections, and placed in individual wells of six-well Falcon tissue culture plates with DMEM supplemented with 10% FBS, penicillin G sodium, and streptomycin sulfate at 37°C in 5% CO_2_. Cells from multiple donors were pooled, and the cell monolayers used at passage three. For inhibitor analysis, cells were pretreated with PP2 (10 μM) or SB203580 (2, 5, 10 and 20 μM), for 3 hr at 37°C before infection. The cells were exposed to each inhibitor at the same concentrations throughout the infection process. Cell toxicity due to the inhibitors was ruled out by trypan blue exclusion performed on cells treated with inhibitors for the same time at the same concentrations. The protocol for use of corneas from deceased human donors was approved by the University of Oklahoma Institutional Review Board, and conformed to the tenets of the Declaration of Helsinki.

HAdV-19 used in this study was isolated from a human patient's cornea, as previously described [28], and grown in A549 cells, (lung carcinoma cells, CCL 185; American Type Culture Collection, Manassas, VA) in MEM with 2% FBS, penicillin G sodium, and streptomycin sulfate. The Oklahoma State Department of Health confirmed the viral serotype. Virus was purified from A549 cells by ultracentrifugation via CsCl gradient, dialyzed against a 10 mM Tris (pH 8.0) buffer that contained 80 mM NaCl, 2 mM MgCl_2_, and 10% glycerol, titered in triplicate and stored at -80°C.

### Viral infection

Monolayer cells grown to 95% confluence in six-well plates were washed in MEM with 2% FBS, and infected with purified HAdV-19 at a multiplicity of infection (MOI) of 50 or mock infected with virus-free dialysis buffer as a control. Virus was adsorbed at 37°C for 1 hr and then incubated for 1 additional hr before RNA isolation. For protein analysis, cells grown to 95% confluence in six-well plates were serum-starved for 18–24 hr before infection, and lysed at 4 hours post-infection.

### Immunoblot analysis

HAdV-19 and mock infected keratocytes were lysed with chilled cell lysis buffer (20 mM Tris, pH 7.4, 150 mM NaCl, 1 mM EDTA, 1 mM EGTA, 1% Triton X-100, 2.5 mM sodium pyrophosphate, 1 mM β-Glycerolphosphate, 1 mM Na_3_VO_4_, 1 μg/ml Leupeptin, and 1 mM PMSF), and incubated at 4°C for 5 min. The cell lysates were cleared by centrifugation at 21,000 × *g *for 15 min. The protein concentration of each supernatant was measured by BCA analysis (Pierce, Rockford, IL) and equalized. Twenty micrograms of cell lysates were subsequently separated by 10% SDS-PAGE and transferred onto nitrocellulose membranes (BioRad, Hercules, CA) and immunoblotted. The bands were visualized with an enhanced chemiluminescence kit (Amersham, Piscataway, NJ). Densitometric analysis of immunoblots where indicated was performed using ImageQuant 5.2 (Amersham) in the linear range of detection, and absolute values were then normalized to total protein or actin as indicated in figure legends.

### p38 MAPK assay

p38 MAPK activity was determined using the p38 MAPK Assay Kit (Cell Signaling). Briefly, endogenous p38 MAPK was immunoprecipitated from 250 μg of cell lysate with immobilized phospho-p38 MAPK (Thr180/Tyr182) monoclonal antibody overnight at 4°C. The precipitates were washed twice with lysis buffer and twice with kinase buffer (25 mM Tris, pH 7.5, 5 mM β-glycerophosphate, 2 mM DTT, 0.1 mM Na_3_VO_4_, and 10 mM MgCl_2_), and suspended in kinase buffer containing cold ATP (200 μM) and ATF-2 fusion protein. After incubation for 30 min at 30°C, the reactions were stopped with 3 × SDS sample buffer (187.5 mM Tris-HCl pH 6.8, 6% w/v SDS, 30% glycerol, 150 mM DTT, 0.03% w/v bromophenol blue). The proteins were resolved by 10% SDS-PAGE followed by western blot analysis. The membranes were probed with antibodies against phospho-ATF-2 (Thr76). Phosphorylated ATF-2 protein from three different experiments was quantified using densitometer scanning, and the means compared by Student's *t *test for each time point.

### RT-PCR

Total RNA was isolated using TRIzol reagent (Invitrogen, Carlsbad, CA) according to the manufacturer's protocol. RNA concentrations and quality were determined spectrophotometrically. The template, cDNA was synthesized by reverse transcription of the total RNA (2 μg) with Moloney murine leukemia virus reverse transcriptase (Promega, Madison, WI) using an oligo(dT) 15 primer (Promega). The primers used for PCR amplification included: IL-8 (Genbank #: AF385628) forward, 5'GTGTGGGTCTGTTGTAGGGT3'; reverse, 5'CTGTGAGGTAAGATGGTGGC3', which amplified a 481-bp product; GAPDH (Genbank #: X01677) forward, 5'GTCGGAGTCAACGGATTTGGTCGT3'; and reverse, 5'GACGGTGCCATGGAATTTGCCATG3', which yielded a 165-bp product. The PCR reaction was performed on Mastercycler^® ^(Eppendorf, Hamburg, Germany) using the following cycling parameters: 94°C for 2 min, 30 cycles of 94°C for 15 sec, 55°C for 15 sec and 72°C for 45 sec, followed by the final step of 72°C for 1 min. The amplification products were analyzed by gel electrophoresis on 1% agarose gel.

### Immunoprecipitation

Whole-cell lysates from infected primary keratocytes (300 μg) were precleared with protein A-Sepharose beads for 30 min. Precleared protein extracts were added to anti-p38 MAPK (Santa Cruz Biotechnology) or isotype control (anti-rabbit) antibodies in phosphate-buffered saline containing protease inhibitors (phenylmethylsulfonyl fluoride [5 × 10^-5 ^M], leupeptin [1 × 10^-2 ^mg/ml], aprotinin [5 × 10^-3 ^mg/ml], and sodium vanadate [30 mM]), 0.1% Tween 20 and rocked at 4°C for 2 h before the addition of protein A-Sepharose (25 μl; 1:1 slurry) and further incubated at 4°C for 12 h. Immunoprecipitates were washed five times with wash buffer (100 mM Tris-Cl pH 8.0, 500 mM NaCl, 0.1% Tween 20) containing protease inhibitors, and proteins were eluted by the addition of sodium dodecyl sulfate (SDS)-polyacrylamide gel electrophoresis sample buffer and boiling for 5 min. Samples were run on 10% SDS-polyacrylamide gels using standard protocols and transferred to nitrocellulose membranes (BioRad). The membrane was probed with anti-phospho-NFκB-p65 and the bands were visualized with an enhanced chemiluminescence kit (Amersham).

### Electrophoretic mobility gel shift assay

Nuclear extracts from HAdV-19 infected and buffer treated (mock) keratocytes were prepared using Nucbuster kit (Novagen, Madison, WI). Binding and supershift assays were done using Dig Gel Shift kit (Roche, Indianapolis, IN) according to the manufacturer's instructions. Briefly, IL-8 sense and anti-sense oligos encoding specific binding sites for NFκB were synthesized (IDT, Coralville, IA) and annealed. Oligos were then labeled using Dig-ddUTP and terminal transferase for 15 min at 37°C in the labeling buffer. For the assay, 5 μg of nuclear extract, labeled oligo-nucleotide, poly (dI-dC) (1 μg), and poly L-lysine (0.1 μg) were mixed in the binding buffer and incubated at room temperature for 15 min. For competition, 100 molar excess of unlabelled probe was added to the reactions 15 min before the addition of labeled probe. For supershift assay, 1 or 2 μg of NFκB-p65 antibody was added to binding reaction and incubated on ice for 30 min prior to adding the probe. Protein-DNA complexes were resolved in 5% pre-electrophoresed polyacrylamide gel in 0.5× TBE running buffer and then transferred to a nylon membrane (Roche). The membrane was then probed for anti-digoxigenin and the bands were detected by chemiluminescense using a Kodak Image Station 4000R (Rochester, NY).

### Confocal microscopy

Keratocytes grown on slide chambers (Nunc, Rochester, NY) were treated with DMSO or SB203580 (10 μM or 20 μM) for 3 hr and then infected with HAdV-19 or dialysis buffer for 20 min. Cells were partially fixed in 0.05% paraformaldehyde for 10 min, washed in PBS containing 2% FBS, and permeabilized in solution containing 0.1% Triton X-100 for 5 min. After 30 min blocking in 3% FBS-PBS, the cells were incubated in 5 μg/ml of NFκB-p65 primary antibody for 1 hr at room temperature, washed and incubated in Alexafluor-594 conjugated secondary antibody (Molecular Probes, Eugene, OR) for 1 hr more at room temperature. Cells were then washed, fixed in 2% paraformaldehyde, and mounted using Vectashield (Vector labs, Burlingame, CA) mounting medium containing DAPI. Images were taken in an Olympus (Center Valley, PA) FlouView 500 confocal microscope using a 60× water immersion objective.

### ELISA

Keratocytes were treated with DMSO or SB203580 (2, 5, 10, and 20 μM) for 3 hr before infection with purified HAdV-19 or virus-free dialysis buffer as a control. The cell supernatants were collected 4 hr post-infection, and the levels of IL-8 quantified by sandwich ELISA. The detection limit was 30 pg/ml. Plates were read on a SpectraMax M2 microplate reader (Molecular Devices, Sunnyvale, CA) and analyzed with SOFTmax analysis software (Molecular Devices). The means of triplicate ELISA values for each of the virus- and mock infected wells were determined, and a dose-response relationship between p38 MAPK inhibitor concentration and IL-8 protein expression examined by linear regression analysis.

### siRNA transfection

Transfections were carried out using Oligofectamine (Invitrogen) following the manufacturer's instructions. Briefly, the transfection mixture was prepared by mixing 12 μl of Oligofectamine to 48 μl of Opti-MEM followed by incubation at room temperature for 5 min, followed by addition of 100 nM SMARTpool p38-MAPK siRNA or control siRNA (Upstate, Charlottesville, VA) and further incubation for 15 min. The transfection complex was added to 50–70% confluence cells, and virus and mock infections carried out 48 hours later. Supernatants and cell lysates were collected 4 hour after infection for IL-8 ELISA and p38 MAPK western blot analysis, respectively. The effect of siRNA on IL-8 ELISA was determined by ANOVA with preplanned contrasts.

## Competing interests

The author(s) declare that they have no competing interests.

## Authors' contributions

JR and XJ participated in the experimental design and performed the experiments for all the data presented in this manuscript, and together provided a first draft of the paper. JR participated in the experimental design and manuscript revision. JC conceived the project, participated in the experimental design and manuscript revision, and is corresponding author. All authors read and approved the final manuscript.
